# Local Accumulation of Lymphocytes in the Intima of Human Aorta Is Associated with Giant Multinucleated Endothelial Cells: Possible Explanation for Mosaicism of Atherosclerosis

**DOI:** 10.3390/ijms23031059

**Published:** 2022-01-19

**Authors:** Nikita G. Nikiforov, Dmitry V. Zlenko, Varvara A. Orekhova, Alexandra A. Melnichenko, Alexander N. Orekhov

**Affiliations:** 1 Laboratory of Medical Genetics, Institute of Experimental Cardiology, National Medical Research Center of Cardiology, 121552 Moscow, Russia; nikiforov.mipt@googlemail.com; 2 Center for Precision Genome Editing and Genetic Technologies for Biomedicine, Institute of Gene Biology, Russian Academy of Sciences, 119334 Moscow, Russia; 3 Laboratory of Angiopathology, Institute of General Pathology and Pathophysiology, 125315 Moscow, Russia; zavod@ifarm.ru; 4 N.N. Semenov Federal Research Center for Chemical Physics RAS, 119991 Moscow, Russia; dvzlenko@gmail.com; 5 Faculty of Biology, M.V. Lomonosov Moscow State University, 119991 Moscow, Russia; 6 Laboratory of Infection Pathology and Molecular Microecology, Institute of Human Morphology, 117418 Moscow, Russia

**Keywords:** giant multinuclear endothelial cells, atherosclerosis, mosaicism, aortic intima, leukocytes, LDL

## Abstract

Distribution of different types of atherosclerotic lesions in the arterial wall is not diffuse, but is characterized by mosaicism. The causes of such distribution remain to be established. At the early stages of atherogenesis, low-density lipoprotein (LDL) particles and immune cells penetrate into the intimal layer of the arterial wall through the endothelium. In adult humans, the luminal surface of the arterial wall is a heterogeneous monolayer of cells with varying morphology including typical endothelial cells (ECs) and multinucleated variant endothelial cells (MVECs). We hypothesized that distribution of MVECs in the endothelial monolayer can be related to the distribution pattern of early atherosclerotic lesions. We obtained en face preparations of intact adult (22–59 years old) aortic wall sections that allowed us to study the endothelial monolayer and the subendothelial layer. We compared the distribution of MVECs in the endothelial monolayer with the localization of early atherosclerotic lesions in the subendothelial layer, which were characterized by lipid accumulation and immune cell recruitment. In primary culture, MVECs demonstrated increased phagocytic activity compared to mononuclear ECs. Moreover, we have shown that unaffected aortic intima contained associates formed as a result of aggregation and/or fusion of LDL particles that are non-randomly distributed. This indicated that MVECs may be involved in the accumulation of LDL in the subendothelial layer through increased transcytosis. Interaction of LDL with subendothelial cells of human aorta in primary culture increased their adhesive properties toward circulating immune cells. Study of unaffected aortic intima revealed non-random distribution of leukocytes in the subendothelial layer and increased localization of CD45+ leukocytes in the subendothelial layer adjacent to MVECs. Together, our observations indicate that MVECs may be responsible for the distribution of atherosclerotic lesions in the arterial wall by participating in LDL internalization and immune cell recruitment.

## 1. Introduction

Atherosclerosis is associated with lipid accumulation in the arterial wall, which is accompanied by the recruitment of inflammatory cells and increased pro-inflammatory signaling. The main source of lipids is circulating low-density lipoproteins (LDLs) penetrating into the arterial wall, while the local inflammatory process is supported by the infiltration of circulating immune cells. Some areas of the vascular system are more prone to atherosclerosis development than others. Notably, areas of disturbed laminar blood flow such as arterial bends and bifurcation points appear to be affected more frequently. Interestingly, even within atherosclerosis-prone areas, atherosclerotic lesions are distributed not diffusely, but locally, and even focally in the arterial wall. The observed mosaic distribution of lesions at different stages of development remains to be explained. Today, it remains unclear why some areas of the arterial wall are susceptible to lipid accumulation and chronic inflammation development, while other adjacent areas do not undergo such changes.

**Figure 1:**
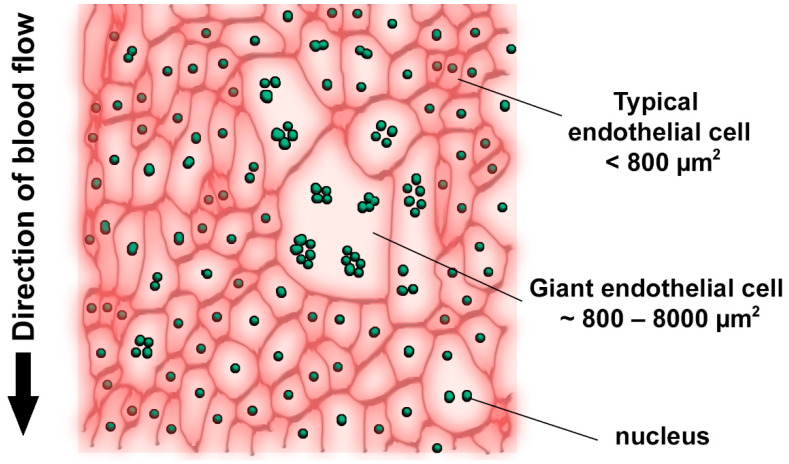
Schematic presentation of the heterogeneous endothelium of the adult human arterial wall [3,4,5].

Modified atherogenic LDL plays a special role in atherosclerosis development, since modified particles have altered uptake and degradation patterns, causing massive intracellular lipid accumulation. LDL particles are also prone to self-association, which further increases their atherogenic properties, although the nature of such LDL “associates” remains to be studied [1,2]. Available studies indicate that reversible aggregation of LDL particles precedes their irreversible fusion. It is clear that, in order to penetrate into the arterial wall intima, both LDL particles and the immune cells should bypass the monolayer of the endothelial cells (ECs) that separates the intima from the arterial lumen. The luminal surface of the arterial wall of adult humans contains heterogeneous cells including typical ECs (less than 800 µm^2^) and giant multinucleated variant endothelial cells (MVECs) (up to 8000 µm^2^) (Figure 1) [3,4,5]. The latter can be observed both in unaffected intima and above the fatty streaks and pronounced atherosclerotic plaques [6,7].

We hypothesized that distribution of the endothelial cells with different morphology can underlie the observed mosaicism of atherosclerotic lesions in the arterial wall. In this study, we aimed to reveal the non-random distribution of cells and molecules involved in early atherogenesis and to characterize the relationship between their localization in the subendothelial intima and the MVECs in the endothelial layer. To that end, we obtained en face preparations from sections of intact aortic wall from adult humans (22–59 years of age). En face preparations allow for study of the endothelial monolayer and the innermost subendothelial layer, which contains 3–5 layers of cells. Such preparations can help study the mutual localization of MVECs and atherosclerosis-associated features such as LDL associates and CD45+ leukocytes.

## 2. Results

### 2.1. Localization of LDL Associates in Unaffected Subendothelial Intima

**Figure 2:**
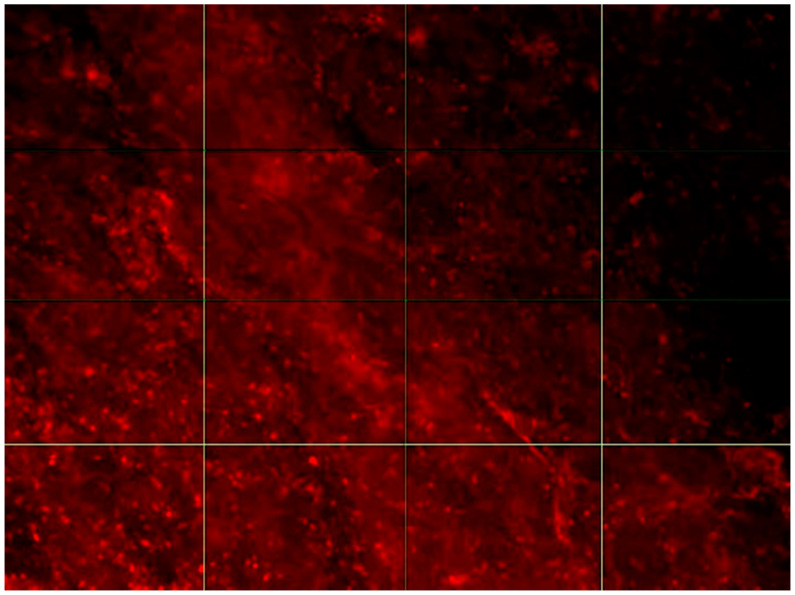
Fluorescence micrograph of LDL associates stained using ApoB-100 antibodies in the aortic intima. Image was taken with a Nikon C1 microscope, 20× lens. The size of the presented area is 100 × 140 µm.

In this study, we did not distinguish between reversible aggregation and irreversible fusion of LDL and did not investigate the nature of LDL association, therefore calling clusters of self-associated LDL particles “LDL associates”. We studied the localization of LDL associates in the subendothelial space of human aortic intima using fluorescent microscopy to examine tissues stained with anti-ApoB-100 antibodies (Figure 2). We particularly focused on LDL associates for the following reasons. Circulating modified LDLs, which induce intracellular lipid accumulation, are prone to spontaneous self-association, which increases the pro-atherogenic properties of modified LDL [8]. It was shown that the affinity of LDL to proteoglycans of the human aorta is dependent on the size of LDL self-associates [9]. The fluorescent microscopy approach that was used in this study allowed for the identification of only relatively large structures (≥1 µm), while the average size of native (non-modified) LDL particles was about 25 nm. Therefore, objects visualized by this method can be considered LDL associates.

The obtained images of the en face preparations demonstrated that the distribution of LDL associates was not random, but included clusters of LDL. We therefore hypothesized that LDL distribution in the subendothelial layer is non-random. To test this hypothesis, we analyzed the images by applying the “index-of-dispersion” method. This model allows for the calculation of the index of aggregation, which reflects the distribution pattern. If the index of aggregation is <1, the distribution can be considered regular. If the index of aggregation is 1, the distribution is random. Such a distribution is homogeneous, without visible clusters. If the index of aggregation >1, the distribution is aggregated, hence, visible clusters are present.

Therefore, calculating the index of aggregation allows for characterization of the randomness of object distribution. Moreover, this method allows for calculating the mean area of clusters in cases of aggregated distribution of the signal.

The parameters of the distribution of LDL associates in the subendothelial layer of the aortic intima of three different subjects are presented in Table 1. In all studied samples, the index of aggregation was found to be >1, indicating the presence of non-random clusters of LDL associates. Typically, the size of such clusters was about 0.1 mm^2^.

The obtained results demonstrated the presence of clusters of LDL associates in the subendothelial layer of human aortic intima.

### 2.2. Localization of CD45+ Leukocytes in the Subendothelial Layer of Unaffected Human Aortic Intima

**Figure 3:**
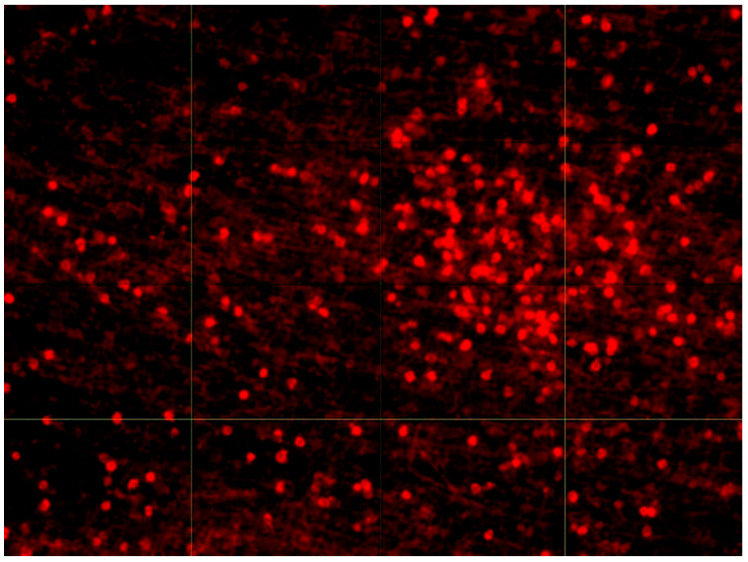
Fluorescence micrograph of CD45+ leukocytes in the aortic intima. Image was taken with a Nikon C1 microscope, 10× lens. The size of the presented area is 390 × 520 µm.

We next tested whether the subendothelial layer of human aortic intima contains clusters of CD45+ leukocytes similar to clusters of LDL associates. Fluorescent micrograph of the intima sample stained with anti-CD45 antibodies is presented in Figure 3.

The parameters of the CD45+ leukocyte distribution in the subendothelial layer of the aortic intima of four different subjects are presented in Table 2. In all studied samples, the index of aggregation was found to be >1, with a typical cluster size of >0.3 mm^2^.

These observations demonstrate the presence of clusters of CD45+ leukocytes in the subendothelial layer of the aortic intima in all studied samples.

### 2.3. Association of MVECs of the Endothelial Layer with Subendothelial Leukocyte Clusters

**Figure 4:**
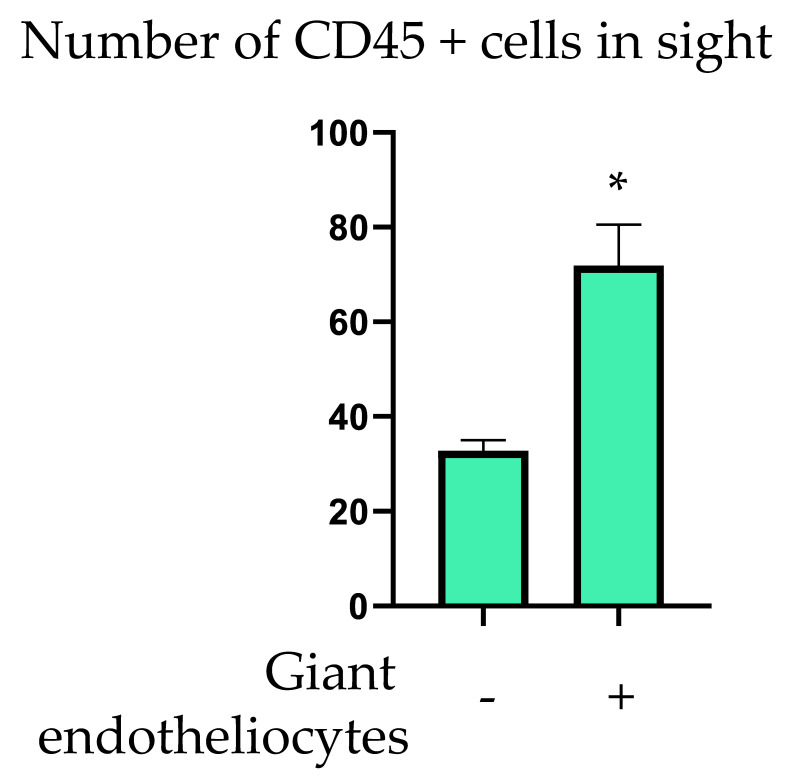
Association of MVECs (giant endotheliocytes) with CD45+ cells in human aortic intima. *—statistically significant difference (*p* < 0.05).

To study the association of MVECs of the endothelial layer with subendothelial CD45+ leukocytes, we performed contrasting staining of the endothelial cells using anti-vimentin antibodies that allowed us to define the cell borders. Subendothelial leukocytes were stained with anti-CD45 antibodies. First, we checked for the presence of MVECs in the field of view of the microscope (100 × 140 µm). The MVECs were detected visually, based on the linear size >50 µm (surface area > 800 µm^2^). We next counted the leukocytes in the present field of view but at a different focus plane, examining three consecutive planes of the subendothelial intima at a distance of 15 µm (as assessed by the position of the focus knob of the microscope). The procedure was repeated for other fields of view. Average leukocyte count per field of view related to the presence or absence of MVEC is presented in Figure 4. In fields of view that contained MVECs, leukocyte count was significantly higher (71.9 ± 8.6 vs. 32.8 ± 2.2, *p* < 0.05).

Our observations demonstrated the presence of clusters of LDL associates in the subendothelial layer of the human aortic intima as well as clusters of CD45+ leukocytes, both types of clusters having a non-random distribution. Typical size of LDL associate clusters was more than 3-fold smaller than that of a typical leukocyte cluster. This difference in observed cluster size brought us to the hypothesis that clusters of LDL associates may precede the CD45+ leukocyte clusters, with potential causative link. To further explore this possibility, we studied the effect of LDL on the adhesive properties of subendothelial cells and their ability to recruit circulating leukocytes.

### 2.4. Effect of LDL on the Adhesive Properties of Subendothelial Cells in Primary Culture

To study the influence of LDL on subendothelial cell adhesion to circulating leukocytes, we obtained primary culture of subendothelial cells of the human aortic intima. After isolation from the tissue sample, subendothelial cells were cultured for seven days in Medium 199 at 37 °C and under 5% CO_2_. After that, cells were incubated with 100 µg/mL LDL for 24 h. Next, cells were cultured together with blood leukocytes added at a concertation of 3 × 105 cells/mL for 2 h. After incubation, cells were washed with sterile PBS and fixed with ice-cold methanol.

**Figure 5:**
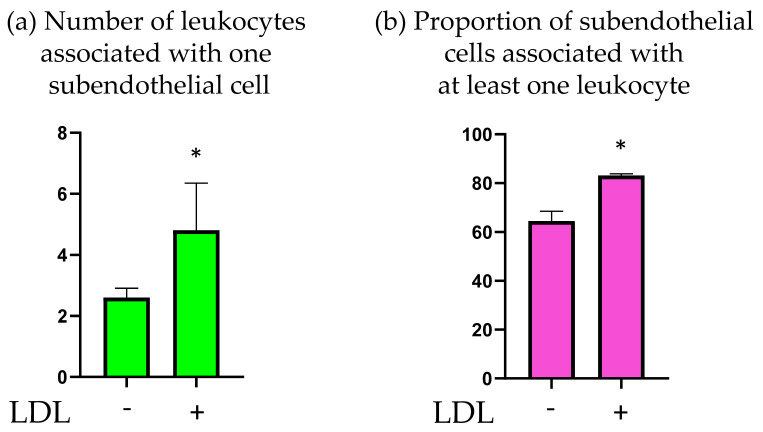
Effect of LDL on the adhesion of human blood leukocytes to intimal subendothelial cells. (**a**) Average number of leukocytes associated with one subendothelial cell. (**b**) The proportion of subendothelial cells associated with at least one leukocyte. *—statistically significant difference (*p* < 0.05).

The obtained samples were stained with anti-CD45 antibodies and the number of leukocytes associated with subendothelial cells was determined. The results are presented in Figure 5. Treatment with LDL led to a significant increase in the mean number of leukocytes associated with subendothelial cells. Moreover, incubation with LDL increased the number of subendothelial cells that were associated with at least one leukocyte.

It can therefore be concluded that incubation of cultured intimal cells with LDL increased their adhesive properties toward circulating leukocytes.

### 2.5. Study of Phagocytic Activity of Mononuclear and Multinuclear Endotheliocytes in Primary Culture

**Figure 6:**
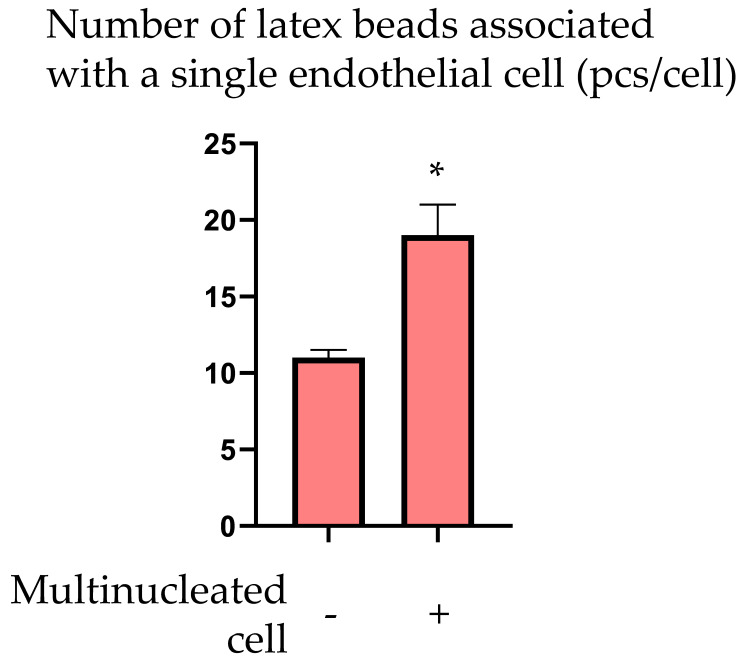
Average number of latex beads associated with one mononucleated or multinucleated EC in culture. *—statistically significant difference (*p* < 0.05).

The cause of the formation of LDL-rich clusters in the subendothelial layer of the intimal wall remains unknown. We hypothesized that typical ECs and MVECs may have different transcytosis activity that can explain the difference in LDL transport from the bloodstream to the subendothelial intima. To test this hypothesis, we assessed the phagocytic activity of the ECs, which corresponds to the initial stages of transcytosis. Since MVEC contain several nuclei, we distinguished them from typical (mononuclear) ECs by this parameter [6]. ECs were isolated from the endothelial monolayer of the human aorta and cultured (seeding density 200,000 cells/cm^2^) for 14–18 days in medium refreshed every other day, until a monolayer was formed. Cells were incubated with 500,000/mL fluorescently labeled latex beads (1.25 µm in diameter, Fluorescent beads, Life Sciences) for 24 h. Afterward, cells were thoroughly washed with sterile PBS at least 10 times to remove free beads, and fixed with ice-cold methanol. Contrasting staining of the nuclei was performed with brilliant green (Sigma-Aldrich, Saint Louis, MO, USA) and toluidine blue (1 mg/mL, Merck KGaA, Darmstadt, Germany). Cells were mounted in glycerin-gelatin mounting medium (Gelatin G2500, Sigma-Aldrich, Saint Louis, MO, USA). Such an approach allowed us to distinguish mononuclear and polynuclear cells and assess the number of fluorescent beads associated with each cell. The results of the comparative analysis of mononuclear and polynuclear ECs are presented in Figure 6. Mean number of fluorescent beads associated with one EC was significantly higher for multinucleated ECs.

The obtained results allowed us to conclude that co-culturing of intimal subendothelial cells and CD45+ blood leukocytes led to their mutual adhesion and that subendothelial cells pre-treated with LDL were associated with higher numbers of CD45+ leukocytes. Furthermore, multinucleated ECs demonstrated increased phagocytosis of latex beads than mononucleated ECs. These in vitro observations indicate that MVEC may perform transcytosis of LDL particles with higher intensity compared to typical ECs. The increased amount of LDL in the subendothelial space adjacent to areas of endothelial lining containing MVECs can stimulate the adhesive properties of subendothelial cells to blood leukocytes, leading to the formation of leukocyte clusters in the subendothelial intima.

## 3. Discussion

Atherosclerotic lesions develop locally and even focally in the arterial wall. Atherosclerotic changes at different stages of development are not diffusely distributed along the arterial wall surface, but demonstrate a certain mosaicism, which remains to be explained. We hypothesized that MVECs may be involved in the early atherogenesis events, and their distribution in the endothelial layer of the arterial wall can underlie the mosaic distribution of atherosclerotic lesions. To test this hypothesis, we studied the spatial association of MVECs with early atherogenic changes in the subendothelial layer such as lipid accumulation and immune cell recruitment.

It has been previously demonstrated by microscopic studies that MVEC may have increased capacity for LDL cholesterol transport and uptake [10]. In this study, MVECs in primary culture were characterized by increased phagocytic activity compared to mononuclear ECs, which was completely in line with the results of the earlier studies. Moreover, we demonstrated that unaffected areas of aortic intima contained LDL accumulation areas that were non-randomly distributed. This allowed us to speculate that MVECs may have increased transcytosis activity, which promotes the accumulation of LDL in the subendothelial layer. Interaction of LDL particles with subendothelial cells in primary culture increased their adhesive properties toward blood leukocytes. Study of unaffected human aortic intima demonstrated that the subendothelial layer also contained clusters of leukocytes distributed in a non-random manner. Finally, a study of spatial association of MVECs and CD45+ leukocytes revealed the association of MVECs present in the endothelial layer and leukocyte clusters located in the adjacent subendothelial areas.

It is currently well recognized that arterial ECs are morphologically heterogeneous in terms of size, protein concentration, and ploidy [7,11,12]. Moreover, the ECs tend to form clusters, similar in size and morphological features. Presence of EC clusters with uniform appearance and distinct borders may indicate that clonal populations of ECs are formed in the arterial wall endothelium. Moreover, since the central area of atherosclerotic plaque is usually covered by homogeneous endothelium, it is possible that cellular populations of the endothelium are replaced by other populations during atherosclerosis development. Interestingly, clustered endothelium development is accompanied by the increase in MVEC number [13]. Clusters of MVECs were observed in atherosclerotic lesions [3]. Several authors have demonstrated that the number of MVECs correlated to the severity of atherosclerotic changes [3,6]. Noteworthy, MVECs could not be detected in aorta samples from children, but their presence was found to increase with age, especially after 30–40 years of age [6]. This may position MVEC as an end product of endothelial histogenesis, or terminal stage of endothelial differentiation and aging. However, exact mechanisms of MVEC formation are not fully understood. Among the possible ways are altered mitosis (with defective cytoplasm fission after daughter nuclei formation) or fusion of several mononuclear cells [14]. Physiological functions of MVEC also remain obscure. On one hand, increase in nuclei number may protect from chromosomal aberrations, and can therefore be regarded as an adaptive mechanism. On the other hand, however, a reduced cell cycle period of multinuclear cells may accelerate the replacement of defective ECs and tissue repair.

Little is known about the possible interaction of MVECs with LDL particles and their transcytosis activity. Internalization of LDL particles by the ECs is primarily mediated by the LDL receptor (LDLR) and toll-like receptor (TLR)4 [15]. It was demonstrated that MVEC expresses more LDLR than typical ECs and participates in LDL uptake more actively [4]. The role of scavenger receptors in LDL transcytosis by the ECs is also known. Endothelial expression of scavenger receptor class B type 1 (SR-BI) was shown to be directly linked to the extent of LDL uptake by human ECs and could be modulated by depletion or overexpression of high mobility group box 1 (HMGB1), which regulates SR-BI expression [16]. In the present study, multinuclear ECs demonstrated enhanced phagocytosis of latex beads compared to mononuclear ECs. However, we did not study the transcytosis activity specifically. Moreover, there are no published data linking MVECs with LDL accumulation in the adjacent subendothelial space. We did not study the mechanisms of such accumulation in the present study, however, the observations made so far allow us to hypothesize that MVECs promote the penetration of LDL particles into the arterial wall intima. Selective increase in transcytosis in MVECs would lead to a non-uniform distribution of LDL associates in the subendothelial space. The non-random distribution of LDL clusters revealed in this study and non-random distribution of LDL-containing particles in early atherosclerotic lesions [17] support this hypothesis.

It is known that circulating LDL particles undergo multiple chemical modifications [18]. Unlike native LDL particles, multiply-modified LDL can induce intracellular lipid accumulation in cells, hence, are atherogenic [8]. Moreover, modified LDL is capable of spontaneous self-association, leading to the formation of large LDL associates (about 1 µm in size), with further increases in atherogenicity [19,20]. Furthermore, an increase in the size of LDL associates improves their affinity to proteoglycans of the arterial intima, therefore promoting their retention in the arterial wall [9,21]. Previous studies have isolated multiply-modified LDL from the blood of atherosclerosis patients [22,23]. Moreover, sialidase activity detected in the blood of atherosclerosis patients may prove to be a triggering mechanism for a cascade of multiple modifications of LDL particles in atherosclerosis [24]. These observations indicate that LDL modification can already be initiated in the circulation. However, further LDL modification can take place in the arterial wall. 

Therefore, it is reasonable to suggest that the two factors that are necessary and sufficient for the development of lipid-enriched areas in the arterial wall are: (1) atherogenic modification of circulating LDL; and (2) local increase in the endothelial permeability for LDL particles. Since the first factor does not explain the spatial distribution of lipid deposits in the arterial wall, it is safe to assume that the formation of areas with increased transcytosis activity can lead to the formation of clusters of LDL associates in the subendothelial layer. Subendothelial accumulation of LDL can further lead to the recruitment of circulating immune cells. Study of areas of diffuse thickening of aortic intima demonstrated a direct correlation between the lipid content and the number of immune cells [17]. It was also demonstrated that the interaction of ECs and MVECs with LDL particles led to increased secretion of cell adhesion molecules, thereby enhancing the monocyte migration [25]. It can be concluded that the results of the current study are well-aligned with the current understanding of early atherogenesis processes.

Increased phagocytic activity of MVECs and their association with clusters of immune cells indicates that MVEC formation may underlie the mosaic pattern of atherosclerotic lesion distribution in the arterial wall. However, the causes and physiological significance of MVEC formation are poorly understood. Since MVECs can act as triggers of local lipid and immune cell accumulation in unaffected intima, their formation may represent one of the earliest events in atherogenesis. Further studies of MVECs may open new opportunities for the prevention and treatment of atherosclerosis. The results of the current work allow us to hypothesize that the following chain of events takes place during atherosclerotic lesion development: (1) MVEC formation; (2) formation of multiply-modified LDL in the bloodstream; (3) increased infiltration of modified LDL in the subendothelial layer of the arterial intima facilitated in the areas of increased transcytosis activity of the endothelium, which hypothetically can be associated with MVEC; (4) formation of LDL self-associates and lipid accumulation in the subendothelial layer; (5) recruitment of circulating leukocytes by the subendothelial cells; (6) formation of lipid-enriched inflammation site beneath the endothelial layer of the arterial wall, which may correspond to the location of MVEC. Further studies should investigate the possible association of MVEC present in the endothelial layer and increased transcytosis and subendothelial LDL accumulation sites, although in vitro studies strongly indicate such a possibility.

## 4. Materials and Methods

### 4.1. En Face Preparations

The study protocol was approved by the Institute for Atherosclerosis Research (Moscow, Russia). En face preparations were made from samples of autopsy material of adult human aorta collected not later than 24 h post-mortem from subjects aged 20 to 59 years (Table 3).

After mechanical removal of adventitia under sterile conditions, pieces of aorta were cut longitudinally and rinsed in Medium 199 containing 100 U/mL of penicillin and streptomycin and 2.5 µg/mL of fungizone (GIBCO, Grand Island, NY, USA). Sections of visually unaffected intima (without visible atherosclerotic changes) were selected for future work. Aortic sections 1.0–1.5 cm in size were rinsed in medium, and thin films of tissue were peeled off from the luminal side. The thickness of the en face samples was 30–50 µm. The obtained en face preparations were fixed with 4% formaldehyde (EMD Millipore, Burlington, MA, USA) for 10 min at +25 °C, rinsed in 0.05% Tween water solution (Tween 20, polyoxyethylene sorbitan monolaurate, SERVA Electrophoresis GmbH, Heidelberg, Germany) and stored at +4 °C.

### 4.2. Immunohistochemistry and Immunofluorescent Staining

The following primary antibodies were used for staining of en face preparations and cultured cells: mouse anti-Human CD45 (Biogenesis, Seattle, WA, USA), mouse anti-Human CX43 (Biogenesis, Seattle, WA, USA), mouse anti-Human Apo B-100 (IMTEK, Moscow, Russia), and mouse anti-Human vimentin (Dako Cytomation, Glostrup, Denmark).

En face preparations fixed in 4% formaldehyde were further fixed with ice-cold (−20 °C) methanol for 30 min. Cell cultures were fixed with ice-cold methanol for 10 min, without prior fixation with 4% formaldehyde. After fixation, samples were rinsed thrice with 0.05% Tween water solution.

To neutralize the endogenous tissue peroxidase, samples were treated with 3% hydrogen peroxide for 30 min at +25 °C and rinsed thrice with 0.05% Tween water solution. Samples were incubated with primary antibodies for 24 h at +4 °C (for en face preparations) or for 1 h at +25 °C (for cell cultures). After the staining, the samples were rinsed thrice with 0.05% Tween water solution.

Biotinylated anti-mouse antibodies (made in Horse, Vector Laboratories, Burlingame, CA, USA) were used as secondary antibodies. Samples were incubated with secondary antibodies for 10–12 h at +4 °C (for en face preparations) or for 3 h at +25 °C (for cell cultures).

The following reagents were used as fluorescent conjugates: Texas Red Streptavidin (TR-S), Fluorescein Streptavidin (Fl-S), and Vectastain ABC reagent (ABC) (all reagents from Vector Laboratories, Burlingame, CA, USA). En face preparations and cell culture samples were incubated with fluorescent reagents TR-S or Fl-S for 30 min at +25 °C, after which the samples were washed with 0.05% Tween water solution. For immune-enzyme staining, en face preparations were incubated for 60 min, while cell culture samples for 30 min at +25 °C. After washing with 0.05% Tween water solution, en face preparations were incubated in 1.5 mL of DAB/Tris-HCl (pH 7.6) (3′3-diaminobenzidine tetrahydrochloride, Amersham International pls., Amersham, UK) for 20 min at +37 °C. After that, 1.5 µL of 30% H_2_O_2_ was added, and the samples incubated for a further 10 min. After the desired staining intensity was achieved, samples were washed with 0.05% Tween water solution.

The following contrasting agents were used for fluorescent microscopy: ethidium bromide (EB), acridine orange (AO), and primuline (all from Sigma-Aldrich, Saint Louis, MO, USA). En face preparations were stained with a water solution of EB (10^−8^ M) and primuline (10^−5^ M). Cultured cells were stained with EB (10^−5^ M), AO (10^−5^ M) and primuline (10^−4^ M). Immuno-enzyme staining was performed using brilliant green (Sigma-Aldrich, Saint Louis, MO, USA) and toluidine blue (1 mg/mL), both dyes used for contrast of the nuclei. Staining with brilliant green was performed for 10 min at +25 °C, and differentiated consecutively with 70% and 96% ethanol. Staining with toluidine blue was performed for 5–10 min at +25 °C and differentiated with 70% or 96% ethanol.

After contrasting, samples were mounted in hydrophilic or hydrophobic mounting medium. Water-containing samples were mounted in hydrophilic gelatin solution (Gelatin G2500, Sigma-Aldrich, Saint Louis, MO, USA) in glycerin. Samples containing alcohols were mounted in Poly-mount (Polysciences Inc., Warrington, PA, USA).

### 4.3. Fluorescent Microscopy

**Figure 7:**
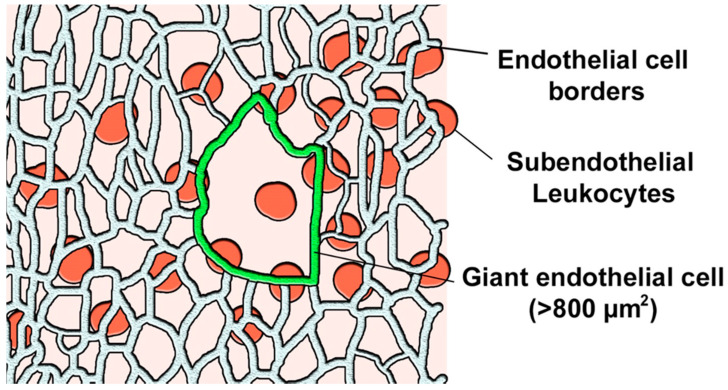
Identification and counting of CD45+ leukocytes in the subendothelial intima co-localized with a giant endothelial cell (marked in green) in one field of view—a schematic example.

En face preparations were examined under fluorescent microscope Nikon C1 with a CoolSnapPro camera and 10× and 20× objective lenses. To study co-localization of ECs of different morphology (stained with antibodies to vimentin) and clusters of CD45+ cells, the following approach was used. A field of view with a size of 100 × 140 µm was examined to visually detect giant ECs. The latter were defined as cells having a linear size of 50 µm and more (surface of >800 µm^2^) (Figure 7).

If a field of view contained at least one giant EC, it was marked as positive (+); if all ECs had regular morphology, they were negative (−). Next, CD45+ leukocytes were counted in the subendothelial layer of the sample in three consecutive sections 15 µm apart, and average numbers of CD45+ cells were calculated.

### 4.4. Primary Culture of the ECs and Subendothelial Cells of Human Aortic Intima

First, ECs were isolated from the autopsy material under sterile conditions. After mechanical removal of the adventitia, the aorta was cut longitudinally and washed in Medium 199 containing 100 U/mL of penicillin and streptomycin and 2.5 µg/mL fungizone (all from Gibco, Grand Island, NY, USA). After washing, the aorta was placed in medium containing 0.08% dispase (Boehringer, Ingelheim, Germany) and incubated for 2 h at +37 °C. After that, the sample was vortexed. Medium containing the suspension of ECs was removed, and the aorta was washed with the fresh medium, which was later combined with the obtained suspension.

The obtained suspension of the ECs was centrifuged at 2000× *g* (3000 rotations per min, Rpm) for 15 min (Beckman TJ-6 centrifuge, Beckman Division, Indianapolis, IN, USA). The pellet was resuspended in MEM medium (Flow Laboratories, Oldham, UK) supplemented with the same antibiotics, heparin (Fluka Chemie AG, Buchs, Switzerland), and endothelial growth factor (EGF, Gibco, Grand Island, NY, USA). Cells were counted and seeded in plastic 35 mm culture plates with a density of 200,000 cells per cm^2^.

After enzymatic removal of the endothelium, subendothelial intimal cells were isolated by collagenase treatment. To this end, pieces of aorta without visible atherosclerotic lesions were selected, and the intimal layer was separated from the media. The obtained material was mechanically (with the aid of forceps) dispersed. The resulting intimal tissue was treated with 0.15% collagenase II (Worthington Diagnostic System, Lakewood, NJ, USA) in Medium 199 (10 mL of enzyme solution per 1 g of tissue), supplemented with 10% fetal bovine serum (FBS, Flow Laboratories, Oldham, UK), 2 mM of L-glutamine, 100 U/mL of penicillin, 100 U/mL of streptomycin, and 2.5 µg/mL of fungizone. Samples were incubated at +37 °C with constant stirring (50 Rpm) in a water bath (New Brunswick Scientific Company, Enfield, CT, USA) until complete disintegration of the tissue (typically for 2–3 h). The obtained suspension of cells was filtrated through nylon filters and centrifuged at +4 °C for 20 min at 1000 rpm. The pelleted cells were washed in 10 mL of Medium 199 supplemented with antibiotics and 10% FBS, and the centrifugation step was repeated. The pellet was resuspended in 10 mL of culture medium, and cells were counted and seeded at a density of 2–4* 104 cells per cm^2^. 

After seeding, ECs and subendothelial cells were cultured at +37 °C under 5% CO_2_ with culture medium refreshed every other day. All experiments were performed on a 7–10-day primary culture of subendothelial cells or 14–18-day culture of ECs that formed a monolayer.

### 4.5. Assessment of Phagocytic Activity

Cells were incubated for 24 h with fluorescent beads 1.25 µm in diameter (Fluorescent beads, Life Sciences, Hercules, CA, USA), final concentration of 500,000/mL. After incubation, cells were thoroughly washed (at least 10 times) with sterile PBS and fixed in ice-cold methanol. Cells were stained with fluorescent dyes as described in Section 4.2. Samples were washed and mounted in aqueous mounting medium (Gelatin, G2500, Sigma-Aldrich, Saint Louis, MO, USA).

### 4.6. Treatment with LDL

Culture medium was refreshed with Medium 199 containing 10% blood serum of the healthy donor’s poor in lipid content obtained by ultracentrifugation (d > 1.215 g/mL). LDL was added to the medium to a final concentration of 100 µg/mL, as assessed by protein content. Control cells were incubated in identical medium without added LDL or serum. After a 24-h incubation, cells were washed twice in PBS and twice in PBS containing 0.2% bovine serum albumin (BSA). Finally, cells were washed three more times in PBS.

### 4.7. Assessment of Non-Random Distribution

To distinguish random and non-random (clustered) distribution of objects in a field of view, we used a method called “index-of-dispersion”. One of the parameters used in this method is the index of aggregation. If the index of aggregation is equal to 1, the distribution can be considered random; if <1, there is a tendency to regular distribution, and if >1, the distribution can be considered non-random (clustering). The index can change depending on the frame size: if the size is such that the dispersion to expectation ratio is maximized, it is optimal for the evaluation of the parameters of distribution, since the size is comparable to the median size of one cluster [26]. 

Therefore, the method of distribution randomness assessment included the following steps: (1) Imaging and identification of single objects (CD45+ leukocytes etc.); (2) division of the obtained image to equal rectangular areas (by 2, 4, 6, 12 parts, etc); (3) calculation of the index of aggregation for all rectangles; and (4) definition of the maximal value of the index of aggregation and the typical cluster size equal to the rectangular area that provided for the maximal index of aggregation.

### 4.8. Statistical Analysis

Data were presented as mean ± standard deviation. Differences between groups in the case-control studies were identified by paired sample T-test using the 21.0 IBM SPSS Statistics for not less than three independent experiments. Graphs were plotted in GraphPad Prism 8.

## 5. Conclusions

In this study, we revealed a relationship between the distribution of MVECs in the endothelial layer and clusters of leukocytes in the adjacent subendothelial layer of the human aortic intima. Although a causative link between MVEC localization and the formation of sites of lipid accumulation leading to atherosclerosis remains to be established by future studies, our work provides important indications of such a connection. Increased phagocytic activity of MVECs may indicate enhanced transcytosis of LDL particles in the corresponding areas, contributing to the self-association of LDL particles and increased adhesiveness of the subendothelial cells to blood leukocytes. Therefore, MVEC formation can potentially serve as a trigger of early atherogenesis events. Further studies should focus on characterizing the physiological significance and mechanisms of MVEC formation, and will help improve our understanding of atherosclerotic lesion formation to develop better therapeutic approaches for atherosclerosis prevention and treatment.

## Figures and Tables

**Table 1:** Distribution parameters of LDL associates in the subendothelial layer of the human aortic wall.

Sample	Index of Aggregation	Average Area of Clusters (mm^2^)
Sample_01	47.9 ± 5.9	0.121 ± 0.062
Sample_05	41.0 ± 5.6	0.084 ± 0.024
Sample_06	125.3 ± 26.9	0.073 ± 0.009

**Table 2:** Distribution parameters of CD45+ leukocytes in the subendothelial layer of the human aortic wall.

Sample	Index of Aggregation	Average Area of Clusters (mm^2^)
Sample_01	20.5 ± 2.2	0.696 ± 0.164
Sample_03	19.6 ± 1.9	0.385 ± 0.027
Sample_05	68.6 ± 10.8	0.742 ± 0.007
Sample_06	30.4 ± 4.6	>0.9

**Table 3:** Characteristics of the autopsy samples of human aortas.

Case N	Age	Gender	Cause of Death	Pathologic Diagnosis
Sample_01	55	f	Acute myocardial infarction	Macrofocal atherosclerosis
Sample_02	53	m	Acute myocardial infarction	Coronary heart disease, thromboembolism of small pulmonary arteries
Sample_03	56	f	Acute myocardial infarction	Pulmonary heart disease, diffuse microfocal cardiosclerosis
Sample_04	47	m	Pulmonary embolism	Pulmonary artery thromboembolism, macrofocal cardiosclerosis
Sample_05	20	m	Traffic accident	Subarachnoid hemorrhage, hepatic rupture, gastrorrhagia
Sample_06	59	m	Acute myocardial infarction	Diffuse macrofocal cardiosclerosis, cardiohepatic insufficiency
Sample_07	53	m	Pulmonary embolism	Pulmonary artery thromboembolism, right kidney cancer, cancerous cachexia, microfocal atherosclerosis

m = male; f = female.

## Data Availability

The data can be provided upon request.
